# Knowledge and acceptance of malaria vaccine among parents of under‐five children of malaria endemic areas in Bangladesh: A cross‐sectional study

**DOI:** 10.1111/hex.13862

**Published:** 2023-09-03

**Authors:** Mohammad Ashraful Amin, Sadia Afrin, Atia S. Bonna, Md Faisal K. Rozars, Mohammad Hayatun Nabi, Mohammad Delwer H. Hawlader

**Affiliations:** ^1^ Department of Public Health North South University Dhaka Bangladesh; ^2^ Public Health Professional Development Society (PPDS) Dhaka Bangladesh; ^3^ Public Health Epidemiologist, HN & HIV Sector Save the Children Dhaka Bangladesh

**Keywords:** acceptance, children, knowledge, malaria, parents, vaccination

## Abstract

**Background:**

Malaria exists as an endemic in many countries including Bangladesh and the malaria vaccine is not yet available here. The study aimed to assess the level of knowledge and acceptance of the malaria vaccination among the parents of children under the age of five in Bangladesh's malaria‐endemic areas and the sociodemographic, behavioural, and household factors associated with the acceptance and knowledge of the malaria vaccine.

**Methods:**

From January to March 2022, a cross‐sectional study was conducted in all five malaria‐endemic districts of Bangladesh, involving 405 parents of children under the age of 5 who met the inclusion criteria. Multiple logistic regression was used to analyze the factor affecting parents' acceptance and knowledge of malaria vaccination in children under five and other variables.

**Results:**

Majority (54%) of the respondents were mothers. Almost half (49%) of the respondents were aged between 26 and 35 years old and around 90% were from rural areas. A small portion (20%) of the participants were housewives and 46% of them completed primary education. Overall, 70% of the study participants reported that they would accept malaria vaccination independently. About one‐fourth (25%) heard about the malaria vaccine and 48% of them mentioned health professionals as the source of information. Knowledge of malaria vaccination was found associated with residence, income, and family size. Acceptance and knowledge were both associated with residence, education, occupation, income, and family size. In a multivariable analysis, housing structure, house wall, house window, knowledge of malaria, testing for malaria, and being diagnosed with malaria were all associated with knowledge of and acceptance of getting vaccinated against malaria.

**Conclusions:**

The present study highlights the necessity of creating awareness of malaria vaccines in epidemic areas of Bangladesh. This study offers crucial data to develop a policy for a novel malaria vaccine, supporting its adoption in Bangladesh.

**Public Contribution:**

This study was based on interviews. The interviewees were recruited as public representatives from the malaria‐endemic area to assist us in building an understanding of knowledge and acceptance of the malaria vaccine among parents of under‐five children in Bangladesh.

## 1 INTRODUCTION

Malaria is a parasitic infection that spreads to humans through the bites of infected female Anopheles mosquitoes. It is both avoidable and treatable. Malaria affected an estimated 241 million people globally in 2021 and killed 627,000.^1^ Children under five are the most vulnerable to malaria, accounting for 67% of all malaria fatalities globally in 2019.^2^ The majority of cases of malaria in Bangladesh (95%–98%) come from 13 districts in the east and south, with total weighted prevalence rates varying from 3.10% to 3.97%.^2^, ^3^, ^4^, ^5^, ^6^, ^7^ The Chittagong Hill Tracts (CHTs) region is hyperendemic, with an estimated incidence of 11% or more.^8^, ^9^, ^10^ *Plasmodium falciparum* is still the most common cause of malaria in Bangladesh, with a range from 54% to 93%.^5^, ^8^, ^11^, ^12^ Bangladesh has a history of endemic malaria transmission in 13 districts, and approximately 17.5 million people are at risk, although only 27,737 cases were reported in 2016.^13^

Malaria is a significant public health concern in Bangladesh, and while recent progress has been made, the disease burden is very spatially and temporally diverse. An episode of malaria in the Netrakona district in 2005 resulted in 1087 cases and 14 deaths.^14^ In 2004, Chittagong, Netrakona, and Cox's Bazar outbreaks affected 4.1, 2.2, and 1.9 million people, and led to 25, 10, and 168 fatalities, respectively. An outbreak in the Bandarban, Rangmati, and Khagrachari districts in 2002 produced approximately 16,000 clinical cases and 177 deaths.^15^ The ‘Southeast area’ (SeA), notably the CHTs and Cox's Bazar, has historically had the most significant malaria incidence.^16^ Malaria was significantly more common in SeA than Northeast area (NeA) which has previously been demonstrated in different studies.^8^, ^15^, ^17^, ^18^

Many malaria vaccines are now being tested in clinical trials and are intended to yield a better malaria control approach. The RTS, S malaria vaccine candidates undergo clinical testing in three sub‐Saharan African nations.^19^, ^20^ Malaria vaccine development has advanced dramatically over the last two decades. As of 2015, the RTS, S vaccine is the only one authorized; it requires four injections and has limited efficiency.^21^ The RTS, S/AS01 malaria vaccine should be given to children starting at 5 months on a four‐dose schedule to reduce the disease prevalence and associated expenditure.^21^ In locations with seasonal solid malaria transmission or periodic malaria transmission with seasonal peaks, countries may contemplate delivering the RTS, S/AS01 vaccine seasonally, with a five‐dose plan, based on a growing body of research.^22^ Malaria resistance has been reported in most new chemotherapeutic measures given out of vaccines.^23^ These vaccines must be provided to children under five because they are one of the most vulnerable groups suffering from the disease. Their immune systems are still constructed and not as potent, and these vaccines could prevent them from the disease.^24^ A consistent measure of confidence and a baseline for comparison is needed to understand the changing trends of vaccination acceptance over time, which can act as an early warning system for taking essential efforts to avert reductions in vaccine knowledge and acceptance. However, there is a lack of data on vaccination acceptability in low‐and middle‐income countries, primarily in Central and South Asia.^25^ To fill the gap in developing a comprehensive national vaccination programme, this study intends to investigate the public's intention to take malaria vaccine by sociodemographic, clinical, and regional characteristics among Bangladeshi adults living in malaria‐endemic areas. In malaria‐endemic regions, vaccination will reduce the burden of the disease's likelihood of incidence. The community will directly and indirectly benefit. In the endemic regions of Bangladesh, there is still no major study focusing on malaria vaccine awareness and acceptability. In light of recent developments in the realm of malaria vaccine innovation, it is crucial to comprehend the degree of awareness and acceptance of this vaccine in nations that are most vulnerable to this perilous and avoidable diseases. The study aimed to assess the knowledge and acceptance of the malaria vaccine with the associated factors among the parents of under‐five children residing in malaria‐endemic areas in Bangladesh.

## 2 METHODS

### 2.1 Study site

**Figure 1:**
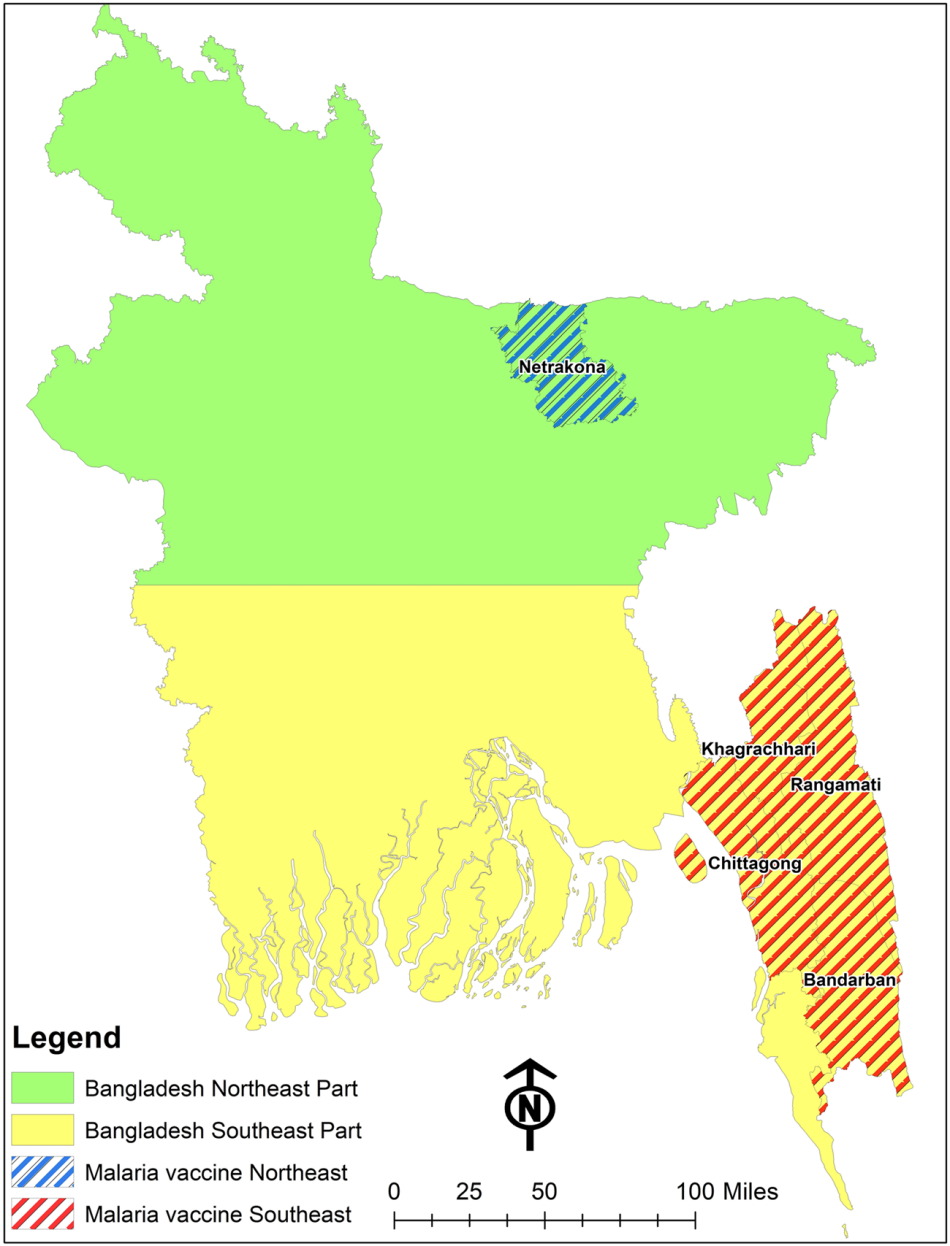
Knowledge and acceptance of the malaria vaccine among parents of under‐five children: northeast and southeast parts of malaria‐endemic areas in Bangladesh.

Bangladesh is divided into eight divisions, 64 districts, 485 upazilas, and 4498 unions.^26^ Eight of the thirteen malaria‐endemic districts are in the northeast and border India, while the remaining five are in the southeast and have a border with both India and Myanmar (Figure 1). This study defines two geographical areas in Bangladesh. The eight malaria‐endemic districts^26^ in the northeast are referred to as ‘the NeA’. We randomly selected one district ‘Netrokona’, as it is one of the malaria hotspot zones of Bangladesh, to make (the NeA) a country representative sample. In contrast, the four malaria‐endemic districts in the southeast are referred to as ‘the SeA’. We collected data from four districts; Bandarban, Chittagong, Khagrachari, and Rangamati. These districts are geographically located in the southeast malaria hotspot zones of Bangladesh.^18^ Due to the higher malaria prevalence in the southeast compared to the northeast, we mainly collected data from four southeast locations. Additionally, due to funding restrictions and limited access, we could not cover more northeastern locations. In terms of population, topography, and malaria risk these places differ (Figure 1). We collected data from the participants, who were selected conveniently as the malaria endemic area was hard to reach.

### 2.2 Study design

We conducted a cross‐sectional study in the malaria‐endemic areas of Bangladesh to determine the knowledge and acceptance of malaria vaccination by parents of under‐five children. We used quantitative methods to collect data.

### 2.3 Sampling and selection

The required sample size was calculated by using a single population proportion formula with an assumption of 95%,^22^ a 5% margin of error, and a 50% proportion of willingness towards the malaria vaccine among caregivers of children under the age of five. The sample size was found to be 384. After adding a 5% nonresponse rate, the final sample size was 405. From January to March 2022, the study was conducted in all five malaria‐endemic districts of Bangladesh, involving 405 parents of children under the age of five who met the inclusion criteria. The inclusion criteria of the participants were; parents older than 18 have at least one under‐five child living in Bandarban, Chittagong, Khagrachari, Rangamat, and Netrokona and have been residents of the area for at least a year. Parents who did not give consent were excluded from the study. Stable malaria hotspots in Bangladesh were divided into two zones: northeast—Netrokona and southeast—Bandarban, Chittagong, Khagrachari, and Rangamati.

A multistage sampling process selected the households in the districts. In the first round of ballots, one community per district was chosen at random. Then, 80 houses were chosen at random using the same procedure to match the study participants. Households were randomly selected in groups of five, with sampling beginning at each community's central place. When a household did not qualify, the next eligible one was chosen. The following step involved selecting homes to take part in the study based on their eligibility. Only residents of the area for at least a year, those over the age of 18, and those with children less than 5 years old were considered for participation. Members of the targeted groups convened at each community centre for a sensitization exercise before the data collection began, during which they were informed about the upcoming household surveys and why their participation was crucial. Community leaders were asked for their input before the visit date was set, and they helped get the group ready.

### 2.4 Ethical considerations

Our study received ethical clearance from the Institutional Review Board/Ethical Review Committee of NSU (2022/0R‐NSU/IRB/0207) and adhered to the 1975 Declaration of Helsinki's ethical criteria (6th version, 2008), as shown in a priori approval by the institutional review committee. All individuals agreed to participate in the study and completed a consent form before the interview.

### 2.5 Data collection instrument

We used a structured questionnaire to collect data for the study. The questionnaire was divided into three domains. The first domain covered socioeconomic factors, the second and third domains covered knowledge and acceptance of malaria infections and vaccinations. A pilot study was carried out with 20 participants to pretest the questionnaire and necessary changes were made. The Bangla translation of the study's English interview questionnaire was vetted by five outside experts (who were not part of the study team and had expertize in the relevant field inside the country, and their feedback and suggestions were considered to improve the quality and validity of the Bangla translation) with fluency in both languages. Once we had the participants' permission and comfort to use their native language Bangla, we went over the interview questions in Bangla. The questionnaires consisted of three sections. Section 1 contained sociodemographic variables such as age, gender (female, male), residence (rural, urban), educational status (no formal education, primary completed, secondary completed, higher), occupation (service holder, business, farmer, day labourer, rickshaw/van puller, boat driver, housewife, unemployed, other), income class (10,000, 10,000–20,000, 20,001–50,000), family size (≤4, ≥5), smoking history and housing structure, malaria‐associated awareness, and associated questions. Sections 2 and 3 contained knowledge and attitudes regarding malaria infection and vaccination. Moreover, the response was recorded as ‘Yes/No’. We calculated each participant's response under the ‘yes’ and ‘no’ categories based on their knowledge and attitude.

### 2.6 Data collection procedure

The study involved training data collectors in data collection methodology, obtaining permission from local leaders in malaria‐endemic areas. Participants were briefed on the study's purpose and malaria vaccine topics, and written informed consent was obtained. Regular reviews were conducted to ensure data quality, and a 20% sample was double‐checked for accuracy. The research was conducted in a malaria‐endemic region, where the local population is exposed to malaria‐related information through government initiatives and stakeholder engagement.

### 2.7 Variables of interest

Several variables were considered explanatory variables based on an extensive literature review and previous descriptive studies.^21^, ^27^, ^28^, ^29^, ^30^, ^31^, ^32^, ^33^, ^34^ The variables relevant to knowledge and acceptance of the malaria vaccine included age, gender, residence, educational status, occupation, income class, family size smoking status, comorbidity, bed net, insecticide‐treated bed net, used insecticide to control mosquitoes, housing structure, house wall, house floor, house window, awareness of malaria, having ever been tested for malaria, ever been diagnosed for malaria. Our dichotomization and cutoff values were theoretical, and a verified approach might have strengthened our findings. In the questionnaire and for the analysis, we added and incorporated those relevant variables. The distribution of responses of the questioners and the goal of distinguishing ‘good’ and ‘poor’ levels led to a cutoff value of 4. The median value above 4 separated the groupings. This method is used in comparable investigations to evaluate and interpret data.

### 2.8 Data analysis

We entered the data into the STATA version 16.0 software. We then calculated each participant's response under the category of ‘yes’ and ‘no’ based on their knowledge and acceptance with the scoring of 0–1. Moreover, the score ranges for both knowledge and attitude were 0–7. Participants with a score of 4 or above were considered to have adequate knowledge and a positive attitude. We developed and modified the questionnaire based on a study in Nigeria.^24^

We used descriptive analysis to describe the independent and dependent variables in terms of frequency and percentage. We described the knowledge and acceptance of the malaria vaccine among the study respondents in proportion.

We categorized acceptance and knowledge scores as good (≥4) and poor (≤3). The choice of a cutoff value of 4 was determined by considering the distribution of responses and aiming to capture a meaningful distinction between ‘good’ and ‘poor’ levels. The median value above 4 was chosen to delineate the two categories. We conducted a bivariate analysis for the associated factors of knowledge and acceptance and measured the association by *χ* ^2^ test. Covariates with a *p* < .20 obtained from the *χ* ^2^ test in the bivariate analysis were included in the multiple regression model. Multiple logistic regression models were fitted to examine the potential adjusted associations between binary outcome data (knowledge and acceptance) and categorical or nominal data (demographic and malaria‐related variables). Exponential coefficients of the binomial logistic regression models were used and presented as odds ratios with a corresponding 95% confidence interval (CI).

All tests were two‐tailed, with a *p* < .05 has considered statistically significant. To measure the internal consistency or reliability of the survey items or questions, Cronbach's *α* with Bootstrap 95% CI based on 1000 samples was used. we found Cronbach's *α* .893 with a 95% CI: 0.879–0.905 for 24 variables which were considered identical questions for malaria risk of infection and vaccination. Again, Cronbach's *α* with 95% CI for the respective seven variables of knowledge and acceptance categories are 0.825 (0.794, 0.85) and 0.949 (0.936, 0.959), respectively. This indicates the items were sufficiently consistent to indicate the measure was reliable.

## 3 RESULTS

### 3.1 Sociodemographic characteristics

Of the 430 people interviewed, 405 responded to the questionnaires (a response rate of 94%). Most respondents (48.89%) were aged between 26 and 35 years, 24.44% were below or equal to 25 years, and 24.69% were between 36 and 50 years old. Among the respondents, 53.33% were female, and 46.67% were male. The majority (89.63%) were from rural areas, and a greater proportion (45.19%) had completed primary education. Almost half (51.85%) were from the Islamic community (Muslim). A total of 19.26% were housewives, 17.78%, 13.83%, 13.09%, and 10.37% were farmers, had a business, service holders, and day labourers, respectively. Most of their family income (48.40%) was below 10,000 (Bangladesh taka [BDT]), 37.28% had income 10,000–20,000 (BDT), and 14.2% had income between above 20,001– 50,000 (BDT). A total of 49.14% of participants had a family size below, or equal to four persons, and 50.86% had ≥5 persons in their family (Table 1).

**Table 1:** Sociodemographic characteristics of the participants (*N* = 405).

Characteristics	Frequency	Percentage (%)
Age group		
≤25	99	24.44
26–35	198	48.89
36–65	108	26.67
Gender		
Male	189	46.67
Female	216	53.33
Residence		
Urban	42	10.37
Rural	363	89.63
Religion		
Islam	210	51.85
Hinduism	31	7.65
Christianity	7	1.73
Buddhism	157	38.77
Educational status		
No Education	43	10.62
Primary completed	183	45.19
Secondary completed	125	30.86
Higher	54	13.33
Occupation		
Service holder	53	13.09
Business	56	13.83
Farmer	72	17.78
Day labourer	42	10.37
Rickshaw/van puller and boat driver	20	4.94
Housewife	78	19.26
Unemployed	24	5.93
Other	60	14.81
Income class (BDT)		
<10,000	196	48.40
10,000–20,000	151	37.28
20,001–50,000	58	14.32
Family size		
≤4	199	49.14
≥5	206	50.86

### 3.2 Geographic distribution

**Figure 2:**
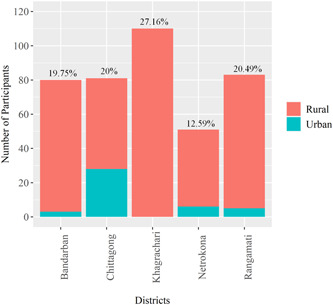
Number of participants in five districts of malaria‐endemic areas of Bangladesh with comparison of urban and rural areas.

Among 405 respondents, 27.16%, 20.49%, 20%, 19.75%, and 12.59% were from Khagrachari, Rangamati, Chittagong, Bandarban, and Netrokona, respectively. In Khagrachari, 100% were from rural areas. Around 90% and 60% of participants were from Bandarban and Chittagong, respectively (Figure 2).

### 3.3 Sources of knowledge about malaria vaccine

**Figure 3:**
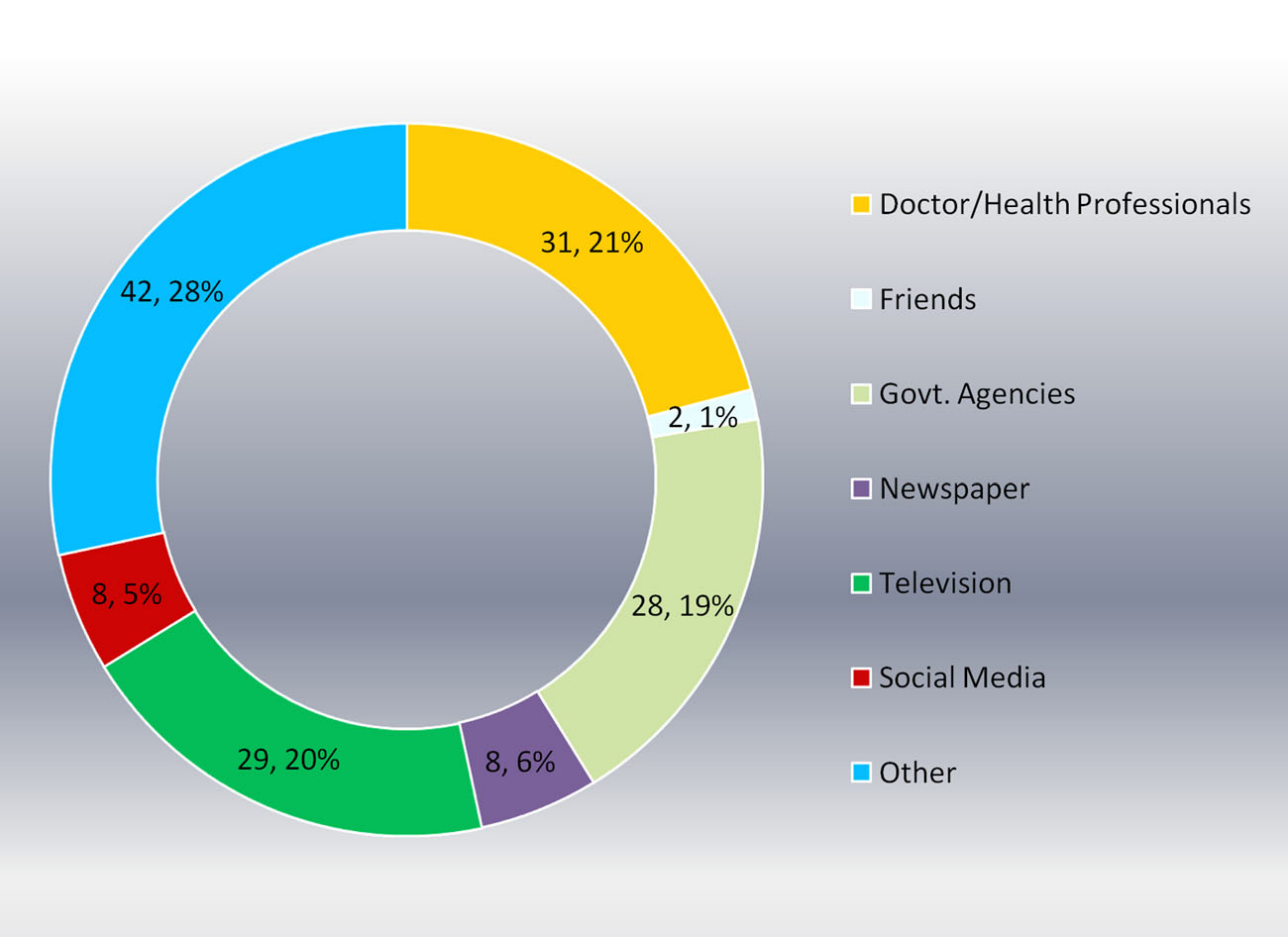
Sources of knowledge about malaria vaccine.

Among the 105 participants who heard about the malaria vaccine, 31.21% learned about it from health professionals. Among all sources, 29.20%, 28.19%, 8.6%, 8.5%, and 2.1% of participants learned about the malaria vaccine from television, government agencies, newspapers, social media, and friends, respectively. The remaining 42.28% of participants learned about the malaria vaccine from other sources (Figure 3).

### 3.4 Knowledge regarding malaria infection and vaccination

The survey revealed that 74.07% of participants were unaware of the malaria vaccine, while 25.93% had just heard about it. Around 69.63% believed malaria was preventable, and 72.10% believed it would minimize man‐hour loss; 63.21% would not hear the World Health Organization (WHO) recommend the vaccine for children in 2021, while 36.79% positively responded. A total of 70% agreed that a malaria vaccine would reduce treatment costs. The participants were questioned once more on their perceptions of the potential bad effects of malaria immunization on their health, and 72.35% of them stated that they do not think such problems will occur as a result of the vaccination (Table 2).

**Table 2:** Knowledge regarding malaria infection and vaccination (*N* = 405).

Questions	Yes, *n* (%)	No, *n* (%)
1. Have you heard of malaria vaccine?	105 (25.93)	300 (74.07)
2. Do you think malaria is preventable with the use of a malaria vaccine?	282 (69.63)	123 (30.37)
3. Do you believe that a malaria vaccine will reduce the number of man‐time lost due to malaria?	292 (72.10)	113 (27.90)
4. Have you heard that the WHO will recommend (RTS, S) malaria vaccine for children in 2021?	149 (36.79)	256 (63.21)
5. Do you believe malaria is a serious health issue that needs vaccination?	292 (72.10)	113 (27.90)
6. Do you agree that a malaria vaccine will reduce the expenditure on treatment?	285 (70.37)	120 (29.63)
7. Do you believe the malaria vaccination will have a negative impact on your health?	112 (27.65)	293 (72.35)

Abbreviation: WHO, World Health Organization.

### 3.5 Acceptance of malaria vaccination

Around 70.86% of respondents stated they would vaccinate their children against malaria, and 69.63% said they wanted to be vaccinated. Approximately 62% of participants desired to spend money on the vaccine, whereas 75.06% wanted it if the government provided it. Around 72.84% thought everyone should get vaccinated. Family size and neighbours would be encouraged to vaccinate their children, according to 74.57% of respondents (Table 3).

**Table 3:** Acceptance of malaria vaccination (*N* = 405).

Questions	Yes, *n* (%)	No, *n* (%)
1. Will you vaccinate your child with malaria vaccine?	287 (70.86)	118 (29.14)
2. Do you want to be vaccinated with the malaria vaccine?	282 (69.63)	123 (30.37)
3. Will you spend money to receive malaria vaccine?	251 (61.98)	154 (38.02)
4. Will you take the malaria vaccine if it is given to you by the government?	304 (75.06)	101 (24.94)
5. Should everyone receive malaria vaccine?	295 (72.84)	110 (27.16)
6. Will you encourage your other family members and neighbours to vaccinate their children against malaria?	302 (74.57)	103 (25.43)
7. Do you think the newly discovered WHO recommend (RTS, S) malaria vaccine for children in 2021 is safe to take?	293 (72.35)	112 (27.65)

Abbreviation: WHO, World Health Organization.

Northern Bangladesh has 84.3% and 92.2% better knowledge and acceptance of malaria infection and vaccine compared to southern Bangladesh, with a significant difference observed, where the p‐value indicates a significant difference in the knowledge and acceptance category between the northern and southern parts of Bangladesh (Table 4).

**Table 4:** Contingency table for the knowledge and acceptance category regarding malaria infection and vaccination between northern and southern part of Bangladesh.

	Knowledge category		*p*‐Value	Acceptance category		*p*‐Value
	Good	Poor		Good	Poor	
Northern Bangladesh	43 (84.3%)	8 (15.7%)	<.01*	47 (92.2%)	4 (7.8%)	<.01*
Southern Bangladesh	232 (65.5%)	122 (34.5%)		254 (71.8%)	100 (28.2%)	

* Means significant.

We discovered that those who responded with good knowledge had around 95% good acceptance of the malaria vaccine, but those who responded with bad knowledge only had 70% poor acceptance (Table 5).

**Table 5:** Analysis of overlap between knowledge and acceptance among the participants.

Knowledge category	Acceptance category		*p*‐Value^a^
	Good	Poor	
Good	261 (94.9%)	14 (5.1%)	<.0001
Poor	40 (30.8%)	90 (69.2%)	

^a^ Pearson's *χ* ^2^ test.

According to our study's findings, the *p*‐value was found to be significant in knowledge of the malaria vaccine factors with sociodemographic; in the cases of residence (where urban had 91% good knowledge), educational status (where higher education had 82% good knowledge), occupation (where businessman had 82% good knowledge), Income (where income 20,001–50,000 had 88% good knowledge). Again, *p*‐value was found to be significant in acceptance of the malaria vaccine with sociodemographic; in the cases of residence (where urban had 92% good knowledge), educational status (where higher education had 85% good knowledge), occupation (where service holder had 81% good knowledge), Income (where income 20,001–50,000 had 97% good knowledge) (Table 6).

**Table 6:** Association of sociodemographic variables with knowledge and acceptance category regarding malaria infection and vaccination.

Characteristics	Knowledge category				Acceptance category			
	Good (≥4) *n* (%)	Poor (≤3) *n* (%)	*p*‐Value	Adj. OR (95% CI)	Good (≥4) *n* (%)	Poor (≤3) *n* (%)	*p*‐Value	Adj. OR (95% CI)
Age (years) group								
≤25	58 (58.6%)	41 (41.4%)	.061	Ref.	67 (67.7%)	32 (32.3%)	.171	Ref.
26–35	138 (69.7%)	60 (30.3%)		1.46 (0.84, 2.54)	154 (77.8%)	44 (22.2%)		1.40 (0.76, 2.57)
36–65	79 (73.1%)	29 (26.9%)		2.07 (1.06, 4.05)	80 (74.1%)	28 (25.9%)		1.48 (0.72, 3.02)
Gender								
Female	141 (65.3%)	75 (34.7%)	.227	–	160 (74.1%)	56 (25.9%)	.903	–
Male	134 (70.9%)	55 (29.1%)		–	141 (74.6%)	48 (25.4%)		–
Residence								
Rural	237 (65.3%)	126 (34.7%)	.002	Ref.	262 (72.2%)	101 (27.8%)	.007	Ref.
Urban	38 (90.5%)	4 (9.5%)		4.07 (1.35, 12.31)	39 (92.9%)	3 (7.1%)		5.31 (1.49, 18.93)
Educational status								
No education	22 (51.2%)	21 (48.8%)	.003	Ref.	24 (55.8%)	19 (44.2%)	.001	Ref.
Primary completed	116 (63.4%)	67 (36.6%)		1.67 (0.82, 3.43)	129 (70.5%)	54 (29.5%)		1.81 (0.85, 3.85)
Secondary completed	93 (74.4%)	32 (25.6%)		2.05 (0.91, 4.65)	102 (81.6%)	23 (18.4%)		2.76 (1.15, 6.59)
Higher	44 (81.5%)	10 (18.5%)		2.45 (0.83, 7.27)	46 (85.2%)	8 (14.8%)		2.28 (0.71, 7.34)
Occupation								
Service holder	41 (77.4%)	12 (22.6%)	.023	Ref.	43 (81.1%)	10 (18.9%)	.012	Ref.
Business	46 (82.1%)	10 (17.9%)		1.50 (0.52, 4.29)	45 (80.4%)	11 (19.6%)		1.01 (0.33, 3.13)
Farmer	43 (59.7%)	29 (40.3%)		1.11 (0.42, 2.91)	58 (80.6%)	14 (19.4%)		3.20 (1.08, 9.49)
Day labourer	22 (52.4%)	20 (47.6%)		0.74 (0.26, 2.10)	21 (50%)	21 (50%)		0.62 (0.20, 1.91)
Rickshaw/van puller and boat driver	13 (65%)	7 (35%)		1.08 (0.31, 3.77)	16 (80%)	4 (20%)		2.04 (0.47, 8.79)
Housewife	52 (66.7%)	26 (33.3%)		1.21 (0.48, 3.10)	55 (70.5%)	23 (29.5%)		1.20 (0.44, 3.32)
Unemployed	14 (58.3%)	10 (41.7%)		1.19 (0.38, 3.78)	17 (70.8%)	7 (29.2%)		2.21 (0.63, 7.81)
Other	44 (73.3%)	16 (26.7%)		1.70 (0.63, 4.60)	46 (76.7%)	14 (23.3%)		1.73 (0.59, 5.10)
Income class (BDT)								
<10,000	116 (59.2%)	80 (40.8%)	<.001	Ref.	133 (67.9%)	63 (32.1%)	<.001	Ref.
10,000–20,000	108 (71.5%)	43 (28.5%)		1.45 (0.87, 2.41)	112 (74.2%)	39 (25.8%)		1.36 (0.78, 2.37)
20,001–50,000	51 (87.9%)	7 (12.1%)		3.38 (1.23, 9.26)	56 (96.6%)	2 (3.4%)		14.26 (2.95, 69.00)
Family size								
≤4	144 (72.4%)	55 (27.6%)	.059	Ref.	160 (80.4%)	39 (19.6%)	.008	Ref.
≥5	131 (63.6%)	75 (36.4%)		0.59 (0.37, 0.94)	141 (68.4%)	65 (31.6%)		0.43 (0.25, 0.72)

Abbreviations: Adj. OR, adjusted odds ratio; CI, confidence interval.

The multiple logistic regression model illustrates that, the participants from urban areas were found to have four times more knowledge (adjusted odds ratio [AOR] = 4.07, 95% CI = 1.35, 12.31) and five times more likely to accept the vaccine (AOR = 1.49, 18.93) compare to the participants from rural areas. In the case of respondents, whose level of education was increasing, the level of education was found to have good knowledge about the malaria vaccine. People earning 20,001–50,000tk had three times more likely to have good knowledge about the malaria vaccine (AOR = 3.38, 95% CI = 1.23, 9.26) and 14 times more likely to have good acceptance of the malaria vaccine (AOR = 14.26, 95% CI = 2.96, 69.00) in compare less than 10,000 taka earners. Participants aged 36–65 had a two times (AOR = 2.07, 95% CI = 1.06, 4.05) good knowledge in compare to ≤25 years. Regarding occupation, there was a significant association between farmers and their acceptance of the malaria vaccine (AOR = 3.20, 95% CI = 1.08, 9.49). Participants with more than or equal to five family size were significantly more likely to accept and also had good knowledge about the malaria vaccine than those with fewer than five family size (AOR = 0.43, 95% CI = 0.25, 0.72) and (AOR = 0.59, 95% CI = 0.37, 0.94) (Table 6).

In a multiple logistic regression models analysis to identify variables linked to the category of knowledge and acceptance of malaria infection and vaccination concerning knowledge about malaria infection and vaccination, the following factors were found to be significantly related: user of the insecticide‐treated, housing structure, wall, floor, and window, knowledge of malaria, and having ever had a malaria test. People using the bed net (70%), lived in stilted house (85%), tin house wall (85%), brick house floor (87%), awareness of malaria (71%), tested for malaria (74%), diagnosed case of malaria (82%) had significantly (*p* < .05) good knowledge about the malaria vaccine. Again, the people using lived in stilted House (86%), tin house wall (88%), brick house floor (100%), tested for malaria (83%), diagnosed case of malaria (92%) had significantly (*p* < .05) good acceptance about the malaria vaccine (Table 7). Factors associated with knowledge were having a house on stilts (AOR = 3.62, 95% CI = 1.23, 10.66) were more likely than their counterparts to be knowledgeable about malaria infection and vaccination. The odds of having knowledge of malaria vaccine were 2.56 times lower in those who used insecticide‐treated bed nets than those who did not use them. People who lived in homes without windows and open walls possessed higher levels of knowledge than those who did not (AOR = 6.50, 95% CI = 2.71, 16.10) and (AOR = 6.52, 95% CI = 2.66, 15.96). Respondents who indicated that they were aware of malaria (AOR = 3.38, 95% CI = 1.61, 7.11) were 3.38 times more knowledgeable about malaria vaccine and infection than those who were not aware. In a multivariable analysis to identify variables linked to the category of acceptance for malaria infection and vaccination, the following factors were discovered to be significantly correlated with the acceptance category: insecticide‐treated bed net, use of insecticide to control mosquitoes, housing elements (housing structure, walls, floors, and windows), knowledge of malaria, and history of malaria testing and also having ever been diagnosed with malaria. Participants who used insecticides to control mosquitoes (AOR = 0.30, 95% CI = 0.14, 0.64) had a 3.33 lower chance of acceptance of the malaria vaccine than those who said no. The likelihood that a person would accept the malaria vaccine was 10.72 and 11.30 times higher in people who lived in homes without windows and open walls (AOR = 10.72, 95% CI = 3.66, 31.41) and (AOR = 11.30, 95% CI = 3.9, 32.72) than in people who lived in homes with closed windows. Participants who checked the boxes for malaria awareness, having ever been tested for malaria, and having ever been diagnosed with malaria were 4.34, 4.53, and 3.50 times more likely to accept the malaria vaccine, respectively: 23.2% (AOR = 4.44, 95% CI = 1.91, 9.83), 16.3% (AOR = 4.53, 95% CI = 2.09, 9.85), and 7.9% (AOR = 3.50, 95% CI = 1.28, 9.60) (Table 7).

**Table 7:** Association of malaria‐related behavioural and household variables with knowledge and acceptance categories regarding malaria infection and vaccination.

Characteristics	Knowledge category				Acceptance category			
	Good (≥4) *n* (%)	Poor (≤3) *n* (%)	*p*‐Value	Adj. OR (95% CI)	Good (≥4) *n* (%)	Poor (≤3) *n* (%)	*p*‐Value	Adj. OR (95% CI)
Smoking status								
No	198 (67.8%)	94 (32.2%)	.949	–	220 (75.3%)	72 (24.7%)	.449	–
Yes	77 (68.1%)	36 (31.9%)		–	81 (71.7%)	32 (28.3%)		–
Comorbidity								
No	231 (67.3%)	112 (32.7%)	.574	–	256 (74.6%)	87 (25.4%)	.733	–
Yes	44 (71%)	18 (29%)		–	45 (72.6%)	17 (27.4%)		–
Bed net								
No	39 (56.5%)	30 (43.5%)	.026	Ref.	51 (73.9%)	18 (26.1%)	.932	–
Yes	236 (70.2%)	100 (29.8%)		2.20 (0.95, 5.12)	250 (74.4%)	86 (25.6%)		–
Insecticide treated bed net								
No	138 (78%)	39 (22%)	<.001	Ref.	147 (83.1%)	30 (16.9%)	<.001	Ref.
Yes	137 (60.1%)	91 (39.9%)		0.39 (0.21, 0.72)	154 (67.5%)	74 (32.5%)		0.70 (0.32, 1.52)
Used insecticide to control mosquito								
No	141 (69.5%)	62 (30.5%)	.501	–	168 (82.8%)	35 (17.2%)	<.001	Ref.
Yes	134 (66.3%)	68 (33.7%)		–	133 (65.8%)	69 (34.2%)		0.30 (0.14, 0.64)
Housing structure								
Ground level house	230 (65.3%)	122 (34.7%)	.004	Ref.	255 (72.4%)	97 (27.6%)	0.026	Ref.
Stilted house	45 (84.9%)	8 (15.1%)		3.62 (1.23, 10.66)	46 (86.8%)	7 (13.2%)		4.40 (1.25, 15.58)
House wall								
Brick	24 (66.7%)	12 (33.3%)	<.001	Ref.	28 (77.8%)	8 (22.2%)	<.001	Ref.
Cement	57 (80.3%)	14 (19.7%)		2.74 (0.88, 8.52)	58 (81.7%)	13 (18.3%)		1.52 (0.4, 5.68)
Tin	71 (85.5%)	12 (14.5%)		7.17 (2.2, 23.32)	73 (88%)	10 (12%)		8.45 (2.04, 35.01)
Wood	13 (65%)	7 (35%)		0.20 (0.04, 1.02)	17 (85%)	3 (15%)		0.22 (0.03, 1.78)
Bamboo	77 (55%)	63 (45%)		0.41 (0.12, 1.39)	88 (62.9%)	52 (37.1%)		0.47 (0.11, 2.08)
Mud	33 (60%)	22 (40%)		0.96 (0.27, 3.44)	37 (67.3%)	18 (32.7%)		1.04 (0.22, 4.84)
House floor								
Cement	106 (82.2%)	23 (17.8%)	<.001	Ref.	114 (88.4%)	15 (52%)	<.001	Ref.
Brick	7 (87.5%)	1 (12.5%)		3.47 (0.27, 44.92)	8 (100%)	0 (0%)		–
Tin	11 (44%)	14 (56%)		0.16 (0.05, 0.54)	12 (48%)	13 (52%)		0.07 (0.02, 0.28)
Wood	17 (81%)	4 (19%)		1.80 (0.42, 7.72)	19 (90.5%)	2 (9.5%)		0.89 (0.12, 6.82)
Bamboo	56 (69.1%)	25 (30.9%)		1.73 (0.56, 5.34)	58 (71.6%)	23 (28.4%)		0.38 (0.10, 1.49)
Mud	78 (55.3%)	63 (44.7%)		0.58 (0.24, 1.43)	90 (63.8%)	51 (36.2%)		0.26 (0.08, 0.80)
House window								
Closable window	147 (60.5%)	96 (39.5%)	<.001	Ref.	155 (63.8%)	88 (36.2%)	<.001	Ref.
No window	42 (73.7%)	15 (26.3%)		6.60 (2.71, 16.10)	50 (87.7%)	7 (12.3%)		10.72 (3.66, 31.41)
Open walls	86 (81.9%)	19 (18.1%)		6.52 (2.66, 15.96)	96 (91.4%)	9 (8.6%)		11.30 (3.9, 32.72)
Awareness of malaria								
No	25 (44.6%)	31 (55.4%)	<.001	Ref.	33 (58.9%)	23 (41.1%)	.005	Ref.
Yes	250 (71.6%)	99 (28.4%)		3.38 (1.61, 7.11)	268 (76.8%)	81 (23.2%)		4.34 (1.91, 9.83)
Ever been tested for malaria								
No	152 (63.6%)	87 (36.4%)	.026	Ref.	162 (67.8%)	77 (32.2%)	<.001	Ref.
Yes	123 (74.1%)	43 (25.9%)		2.17 (1.1, 4.26)	139 (83.7%)	27 (16.3%)		4.53 (2.09, 9.85)
Ever been diagnosed for malaria								
No	192 (63.2%)	112 (36.8%)	<.001	Ref.	208 (68.4%)	96 (31.6%)	<.001	Ref.
Yes	83 (82.2%)	18 (17.8%)		2.27 (1.04, 4.92)	93 (92.1%)	8 (7.9%)		3.50 (1.28, 9.60)

Abbreviations: Adj. OR, adjusted odds ratio; CI, confidence interval.

We separately analyze the essential questions to determine acceptability (would you vaccinate your child with the malaria vaccine) and knowledge (have you heard of the malaria vaccine). Residence, occupation, smoking status, comorbidity, insecticide treated bed net, house wall, house floor and window, diagnosed and tested for the malaria significantly associated with the knowledge about the malaria vaccine and also residence, educational status, occupation, income class. family member, bed net, housing structure, house floor and window, awareness of malaria and diagnosis for malaria significantly associated with the acceptance of the malaria vaccine (Supporting Information: Tables S1 and S2).

## 4 DISCUSSION

Malaria has become one of the most severe public health issues facing worldwide. Malaria claims the lives of hundreds of thousands of people each year, most of whom are children.^22^ The WHO's goal is for the world to be ‘malaria‐free’. However, factors like antimalarial resistance are delaying eradication in Southeast Asia, and malaria vaccine knowledge and acceptance can be an issue. Bangladesh had some of the highest childhood immunization rates in the WHO South‐East Asia Region for multiple antigens, and coverage rose significantly over the 10 years 2004–2014.^35^, ^36^ This is the first research in Bangladesh to assess the knowledge and acceptance rate of a malaria vaccination among parents of children under five living in malaria‐endemic areas.

Periodic, hypoendemic malaria transmission primarily occurred in geographic regions in Bangladesh, persisting over time. High‐risk groups contribute significantly to the overall malaria burden, despite representing a minor percentage of the population.

Vaccination is the most effective method for controlling infectious diseases, but its success is disputed by withholding or denying vaccinations. This raises the likelihood of outbreaks and epidemics due to vaccine reluctance. The development of a malaria vaccine will significantly impact malaria eradication plans.

A study in Nigeria found that information about the RTS, S malaria vaccine was initially obtained from healthcare professionals, similar to our research. In Ethiopia, marital status, knowledge, and childhood vaccination experience were significantly associated with willingness to accept the vaccine but in our study knowledge of malaria vaccination was found associated with residence, income, and family size. In India, males showed higher vaccine awareness than females, with education and child care being associated with vaccine knowledge.

When the malaria vaccine became available, 287 parents (70.8%) said they would be willing to give their children a dose. The results of this study were lower than those of studies conducted in Nigeria's capital city of Abuja (98%) and the cities of Ibadan (87%) and Tanzania (94.5%), Kenya (88%), the rural community in the south east (Mgbogodo, in Nkanu west local government area in Enugu) of Nigeria (91.6%).^37^, ^38^, ^39^, ^40^, ^41^, ^42^ This variation may be brought about by variations in the sociodemographic characteristics of survey respondents, study timeframe, sample size, and study methodology. In this study, respondents answered for the vaccination to be distributed for free, indicating that they might be unable to afford the price or unwilling to pay for the dose. Our study found that 61.9% of parents of children under five were willing to pay for the children's malaria vaccine, which is virtually the same as research done in Ethiopia (60.6%).^43^ A study in Kenya^40^ 88% of participants and 56% of those who had never attended school indicated they would welcome malaria immunization for a baby in their culture and their child. Compared to this study, our participants' acceptance of a malaria vaccine for their child was around 70%, while our answer to not having any education was only 10%. In Ghana,^44^ malaria vaccination preference was 70%, the same as in our study.

Regarding the housing variables, their direct impact on knowledge and acceptance might not be immediately apparent. However, it is possible that housing conditions serve as proxies for broader socioeconomic factors, such as access to education, healthcare services, or information sources. These socioeconomic factors, in turn, could influence individuals' knowledge and acceptance of the malaria vaccine.

Assessing malaria vaccination knowledge and acceptance among parents under 5 years of age can help policymakers effectively educate the community. Understanding sociodemographic factors, common knowledge, challenges, and facilitators can help increase vaccine awareness in malaria‐endemic areas. The WHO aims to reduce malaria case incidence, fatality rates, and eliminate malaria in at least 35 nations by 2030.^1^

Vector management is crucial for malaria control and elimination. Primary therapies include insecticide‐treated nets and indoor residual spraying. Preventive chemotherapy uses drugs to minimize infections. Chemoprophylaxis, intermittent preventive treatment in infants and intermittent preventive treatment of malaria in pregnancy, seasonal malaria chemoprevention, and mass medication administration are part of mass drug administration. Since October 2021, WHO recommends children in areas with moderate to high *P. falciparum* malaria transmission receive the RTS, S/AS01 malaria vaccine.

Bangladesh's achievement of Millennium Development Goal 4 to reduce childhood mortality is mainly due to the vital national commitment to childhood vaccination. Bangladeshi children under the age of five died at a rate of 46 per 1000 live births in 2014, down from 133 per 1000 in 1993.^35^ Bangladesh has put forth a lot of effort in public health to reduce vaccine‐preventable infections in children. Since the last recorded case of polio in 2006, sustained high polio vaccine coverage has rapidly reduced incidents of polio. Bangladesh's government has set a target of eliminating measles by 2018, coinciding with the WHO's South‐East Asia Regional aim of eliminating the disease by 2020.^45^ Despite these achievements, Bangladesh remains one of the world's fastest‐growing countries, with over 162 million people.^46^ In 2020, there were 29.1 fatalities per 1000 live births among children under five.^47^ While Bangladesh has made substantial progress in increasing childhood immunization coverage, more attention to the series completion of all recommended EPI vaccinations is required to achieve even more significant reductions in childhood morbidity and mortality.^48^ Malaria could become a future calamity in malaria‐endemic areas of Bangladesh if we do not consider malaria vaccines for children under the age of five. Childhood immunization coverage was highest among children in Bangladesh, so approximately 25% of respondents heard about the malaria vaccine in this study. However, their acceptance of the malaria vaccine for their children was around 70%, which is quite surprising for only being possible for the highest acceptance vaccination in Bangladesh.

Hence, it is possible for individuals who have no awareness of malaria to still have some knowledge of the vaccine, although limited or general knowledge. Based on our findings, we observed that a considerable proportion of participants expressed acceptance of the malaria vaccine, even in cases where they reported not hearing about it. This suggests that acceptance may not solely depend on awareness or knowledge of the vaccine. Other factors such as trust in healthcare providers, perceived benefits of vaccination, and cultural beliefs could influence individuals' acceptance of the vaccine.

Bangladesh has made significant progress in immunization coverage through the Expanded Programme on Immunization (EPI) programme, focusing on essential vaccines for infants and children. The country has achieved high coverage rates for measles, diphtheria–tetanus–pertussis, and polio, and has reduced child mortality rates. The study aimed to explore factors associated with malaria vaccine acceptance, but acknowledges that high acceptance rates may be a cause of the EPI programme's success. However, we acknowledge that the high acceptance rates of different vaccines in the EPI programme in Bangladesh might be one of the causes of the EPI programme and could provide valuable insights into the acceptance of malaria vaccine patterns among the population.

There are several implications for this study. First, the study was the first in Bangladesh to assess hotspot areas' knowledge of and acceptance of malaria vaccination and its determinants. Second, the study's high response rate (94%) was likely due to participants' interest (especially in malaria‐endemic areas) in the subject matter and the survey's objectives being communicated succinctly and clearly. Criterion‐based sampling approaches were used to achieve the study's objectives. Seven more locations (endemic malaria sites in Bangladesh) were left unexplored due to a lack of funding, investigation, and/or accessibility. Furthermore, while key findings are consistent with those reported in other studies, some findings may be subject to social desirability bias, which means that in some situations, such as when asked about a vaccine that does not yet exist, participants may have been more inclined to give socially desirable responses rather than describe actual beliefs and practices. The results would be more informative if this study used a mixed‐methods approach.

## 5 CONCLUSION

The study found that most parents in Bangladesh's malaria epidemic area are willing to use the malaria vaccine for their children when it is available. Factors such as age group, residence, education level, occupation, and income class influence the knowledge and acceptability of the vaccine. Factors like insecticide usage, house structure, and history of malaria testing also influence vaccination decisions. To ensure the availability of the malaria vaccine, the government should raise public awareness and allocate more funds for malaria preventive programmes.

## AUTHOR CONTRIBUTIONS

**Mohammad Ashraful Amin**: Conceptualization; data curation; formal analysis; investigation; methodology; project administration; software; visualization; writing—original draft preparation; writing—review and editing. **Sadia Afrin**: Data curation; methodology; visualization; writing—original draft preparation. **Atia S. Bonna**: Data curation; methodology; visualization; writing—original draft preparation. **Md Faisal K. Rozars**: Data curation; formal analysis; visualization. **Mohammad Hayatun Nabi**: Methodology; supervision; visualization; writing—original draft preparation, writing—review and editing. **Mohammad Delwer H. Hawlader**: Methodology; supervision; visualization; writing—original draft preparation; writing—review and editing.

## CONFLICT OF INTEREST STATEMENT

The authors declare no conflict of interest.

## Supporting information

Supporting information.

Supporting information.

## Data Availability

The data that support the findings of this study are available on request from the corresponding author. The data are not publicly available due to privacy or ethical restrictions.

## References

[1] World Health Organization . Malaria. October 22, 2022. Accessed June 2, 2022. https://www.who.int/news-room/fact-sheets/detail/malaria

[2] Bailey P , Lobis S , Maine D , Fortney JA . Monitoring Emergency Obstetric Care: a Handbook. World Health Organization; 2009.

[3] Ahmed SM , Haque R , Haque U , Hossain A . Knowledge on the transmission, prevention and treatment of malaria among two endemic populations of Bangladesh and their health‐seeking behaviour. Malar J. 2009;8(1):173. 19640282 10.1186/1475-2875-8-173 PMC2729311

[4] Alam MS , Khan MGM , Chaudhury N , et al. Prevalence of anopheline species and their *Plasmodium* infection status in epidemic‐prone border areas of Bangladesh. Malar J. 2010;9(1):15. 20074326 10.1186/1475-2875-9-15 PMC2841608

[5] Alam MS , Mohon AN , Mustafa S , et al. Real‐time PCR assay and rapid diagnostic tests for the diagnosis of clinically suspected malaria patients in Bangladesh. Malar J. 2011;10(1):175. 21703009 10.1186/1475-2875-10-175 PMC3145608

[6] Haque U , Ahmed SM , Hossain S , et al. Malaria prevalence in endemic districts of Bangladesh. PLoS One. 2009;4(8):e6737. 19707580 10.1371/journal.pone.0006737 PMC2726938

[7] Maude RJ , Dondorp AM , Faiz MA , et al. Malaria in southeast Bangladesh: a descriptive study. Bangladesh Med Res Counc Bull. 2008;34(3):87‐89. 19476253 10.3329/bmrcb.v34i3.1757

[8] Haque U , Sunahara T , Hashizume M , et al. Malaria prevalence, risk factors and spatial distribution in a hilly forest area of Bangladesh. PLoS One. 2011;6(4):e18908. 21533048 10.1371/journal.pone.0018908 PMC3080915

[9] Khan WA , Sack DA , Ahmed S , et al. Mapping hypoendemic, seasonal malaria in rural Bandarban, Bangladesh: a prospective surveillance. Malar J. 2011;10(1):124. 21569599 10.1186/1475-2875-10-124 PMC3112456

[10] Masud Ahmed S . Differing health and health‐seeking behaviour: ethnic minorities of the Chittagong Hill Tracts, Bangladesh. Asia Pac J Public Health. 2001;13(2):100‐108. 12597507 10.1177/101053950101300208

[11] Thriemer K , Matt J , Walochnik J , et al. Indigenous *Plasmodium ovale* malaria in Bangladesh. Am J Trop Med Hyg. 2010;83(1):75‐78. 10.4269/ajtmh.2010.09-0796 PMC2912579 20595481

[12] Hussain SM , Rahman MM , Ahmed Z , Siddique MM . The recent malaria situation in Chittagong, Bangladesh. Southeast Asian J Trop Med Public Health. 2003;34(suppl 2):1‐5. 19238662

[13] Sarker AR , Sultana M . Cost‐effective analysis of childhood malaria vaccination in endemic hotspots of Bangladesh. PLoS One. 2020;15(5):e0233902. 32470101 10.1371/journal.pone.0233902 PMC7259743

[14] Karim MA , Kabir MM , Siddiqui MA , Laskar MSI , Saha A , Naher S . Epidemiology of imported malaria in Netrokona district of Bangladesh 2013‐2018: analysis of surveillance data. Malar Res Treat. 2019;2019:1‐9. 10.1155/2019/6780258 PMC6595392 31312425

[15] Reid HL , Haque U , Roy S , Islam N , Clements AC . Characterizing the spatial and temporal variation of malaria incidence in Bangladesh, 2007. Malar J. 2012;11(1):170. 22607348 10.1186/1475-2875-11-170 PMC3465176

[16] MEDBOX . Malaria National Strategic Plan 2015‐2020 medbox.org. 2022. Accessed June 2, 2022. https://www.medbox.org/document/malaria-national-strategic-plan-2015-2020#GO

[17] Alam MS , Kabir MM , Hossain MS , et al. Reduction in malaria prevalence and increase in malaria awareness in endemic districts of Bangladesh. Malar J. 2016;15(1):552. 27836016 10.1186/s12936-016-1603-0 PMC5105313

[18] Islam N , Bonovas S , Nikolopoulos GK . An epidemiological overview of malaria in Bangladesh. Travel Med Infect Dis. 2013;11(1):29‐36. 23434288 10.1016/j.tmaid.2013.01.004

[19] RTS, S Clinical Trials Partnership . Efficacy and safety of RTS, S/AS01 malaria vaccine with or without a booster dose in infants and children in Africa: final results of a phase 3, individually randomised, controlled trial. Lancet. 2015;386(9988):31‐45. 25913272 10.1016/S0140-6736(15)60721-8 PMC5626001

[20] RTS, S Clinical Trials Partnership . Efficacy and safety of the RTS, S/AS01 malaria vaccine during 18 months after vaccination: a phase 3 randomized, controlled trial in children and young infants at 11 African sites. PLoS Med. 2014;11(7):e1001685. 25072396 10.1371/journal.pmed.1001685 PMC4114488

[21] Asmare G . Willingness to accept malaria vaccine among caregivers of under‐5 children in Southwest Ethiopia: a community based cross‐sectional study. Malar J. 2022;21(1):1‐8. 35549710 10.1186/s12936-022-04164-z PMC9097094

[22] World Health Organization . Q&A on RTS, S malaria vaccine. 2022. Accessed June 2, 2022. https://www.who.int/news-room/questions-and-answers/item/q-a-on-rts-s-malaria-vaccine

[23] Gogoi N , Zaman MK . Clinical trials in malaria. In: Shegokar R , Pathak Y , eds. Malarial Drug Delivery Systems: Advances in Treatment of Infectious Diseases. Springer; 2023:305‐331.

[24] Onyekachi O , Abana CC , Nwajiobi OF . Prevalence of malaria and wiliness to accept malaria vaccine amongst parents, guardians and caregivers of children under 5 years. Asian Res J Curr Sci. 2021;3(1):36‐50.

[25] Abedin M , Islam MA , Rahman FN , et al. Willingness to vaccinate against COVID‐19 among Bangladeshi adults: understanding the strategies to optimize vaccination coverage. PLoS One. 2021;16(4):e0250495. 33905442 10.1371/journal.pone.0250495 PMC8078802

[26] Noé A , Zaman SI , Rahman M , Saha AK , Aktaruzzaman MM , Maude RJ . Mapping the stability of malaria hotspots in Bangladesh from 2013 to 2016. Malar J. 2018;17(1):259. 29996835 10.1186/s12936-018-2405-3 PMC6042289

[27] Aremu TO , Ajibola OA , Oluwole OE , Adeyinka KO , Dada SO , Okoro ON . Looking beyond the malaria vaccine approval to acceptance and adoption in Sub‐Saharan Africa. Front Trop Dis. 2022;3:22.

[28] Chinar S , Aremu TO , Pranjal G , Shah K , Okoro ON . Awareness of the malaria vaccine in India. Cureus. 2022;14(9):e29210. 36262953 10.7759/cureus.29210 PMC9574518

[29] Immurana M , Boachie MK , Klu D , et al. Determinants of willingness to accept child vaccination against malaria in Ghana. Int J Health Plann Manage. 2022;37(3):1439‐1453. 34984733 10.1002/hpm.3406

[30] Kajungu D , Muhoozi M , Stark J , Weibel D , Sturkenboom M . Vaccines safety and maternal knowledge for enhanced maternal immunization acceptability in rural Uganda: a qualitative study approach. PLoS One. 2020;15(12):0243834. 10.1371/journal.pone.0243834 PMC7728220 33301495

[31] Ngadjeu CS , Talipouo A , Kekeunou S , et al. Knowledge, practices and perceptions of communities during a malaria larviciding randomized trial in the city of Yaoundé, Cameroon. PLoS One. 2022;17(11):0276500. 10.1371/journal.pone.0276500 PMC9632894 36327271

[32] Onyekachi O , Abana CC , Favour ON . Prevalence of malaria and wiliness to accept malaria vaccine amongst parents, guardians and caregivers of children under 5 years. Asian J Curr Sci. 2021;3(1):36‐50.

[33] Thellier M , Houzé S , Pradine B , et al. Assessment of electronic surveillance and knowledge, attitudes, and practice (KAP) survey toward imported malaria surveillance system acceptance in France. JAMIA Open. 2022;5(1):ooac012. 35571356 10.1093/jamiaopen/ooac012 PMC9097633

[34] Wagnew Y , Hagos T , Weldegerima B , Debie A . Willingness to pay for childhood malaria vaccine among caregivers of under‐five children in Northwest Ethiopia. Clinicoecon Outcomes Res. 2021;13:165‐174. 33758520 10.2147/CEOR.S299050 PMC7979355

[35] Boulton ML , Carlson BF , Power LE , Wagner AL . Socioeconomic factors associated with full childhood vaccination in Bangladesh, 2014. Int J Infect Dis. 2018;69:35‐40. 29421667 10.1016/j.ijid.2018.01.035

[36] Hossain M , Sobhan M , Rahman A , Flora SS , Irin ZS . Trends and determinants of vaccination among children aged 06–59 months in Bangladesh: country representative survey from 1993 to 2014. BMC Public Health. 2021;21(1):1‐11. 34419002 10.1186/s12889-021-11576-0 PMC8379560

[37] Abdulkadir BI , Ajayi IO . Willingness to accept malaria vaccine among caregivers of under‐5 children in Ibadan North Local Government Area, Nigeria. Malaria World J. 2015;6(2):146. 10.5281/zenodo.10870005 PMC11107874 38779629

[38] Etokidem A , Ndifon W , Asibong U . Perception and acceptability of malaria vaccine among maternal and child health clinic attendees at the University of Calabar Teaching Hospital, Calabar, Nigeria. J Commun Med Prim Healthc. 2015;27(2):51‐58.

[39] Musa‐Booth TO , Enobun BE , Agbomola AJ , Shiff CJ . Knowledge, attitude and willingness to accept the RTS, S malaria vaccine among mothers in Abuja Nigeria. medRxiv. 2021;4(1). https://www.aamronline.org/index.php/aamr/article/view/128

[40] Ojakaa DI , Jarvis JD , Matilu MI , Thiam S . Acceptance of a malaria vaccine by caregivers of sick children in Kenya. Malar J. 2014;13(1):172. 24886650 10.1186/1475-2875-13-172 PMC4022976

[41] Romore I , Ali AM , Semali I , Mshinda H , Tanner M , Abdulla S . Assessment of parental perception of malaria vaccine in Tanzania. Malar J. 2015;14(1):355. 26383545 10.1186/s12936-015-0889-7 PMC4573291

[42] Peter E , Jothi M , Nwagboso C , Ogbudu S , Eze N , Echieh C . Type a interrupted aortic arch with taussig‐bing anomaly: an unusual indication for staged repair. Niger J Med. 2018;27(3):196‐203.

[43] Wagnew Y , Hagos T , Weldegerima B , Debie A . Willingness to pay for childhood malaria vaccine among caregivers of under‐five children in Northwest Ethiopia. Clinicoecon Outcomes Res. 2021;13:165‐174. 33758520 10.2147/CEOR.S299050 PMC7979355

[44] Kasasa S , Asoala V , Gosoniu L , et al. Spatio‐temporal malaria transmission patterns in Navrongo demographic surveillance site, Northern Ghana. Malar J. 2013;12(1):63. 23405912 10.1186/1475-2875-12-63 PMC3618087

[45] Khanal S , Bohara R , Chacko S , et al. Progress toward measles elimination—Bangladesh, 2000–2016. MMWR Morb Mortal Wkly Rep. 2017;66(28):753‐757. 28727678 10.15585/mmwr.mm6628a3 PMC5657944

[46] National Institute of Population Research and Training—NIPORT/Bangladesh, Mitra and Associates, and ICF International Bangladesh Demographic and Health Survey 2014. Dhaka, Bangladesh: NIPORT, Mitra and Associates, and ICF International. 2016.

[47] World Health Organization . Bangladesh, key demographic indicators. June 6, 2022. Accessed June 2, 2022. https://data.unicef.org/country/bgd/

[48] Sarkar PK , Sarker NK , Doulah S , Bari TIA . Expanded programme on immunization in Bangladesh: a success story. Bangladesh J Child Health. 2017;39(2):93‐98.

