# Supplementary material for: Knowledge and acceptance of malaria vaccine among parents of under‐five children of malaria endemic areas in Bangladesh: A cross‐sectional study
Source: Health Expect. 2023 Sep 3;26(6):2630–43. doi: 10.1111/hex.13862 (PMC10632622; doi:10.1111/hex.13862)
Supplement: Supplementary file 1 — Supporting information. [file HEX-26--s002.docx]

### Supplementary Table 1: Association of socio-demographic variables with Knowledge and Acceptance of Malaria Vaccine (N=405)

|  | **Knowledge Category** | | | | **Acceptance Category** | | | |
| --- | --- | --- | --- | --- | --- | --- | --- | --- |
| **Factor** | **Crude OR (95%CI)** | **P Value** | **Adj. OR (95%CI)** | **P Value** | **Crude OR (95%CI)** | **P Value** | **Adj. OR (95%CI)** | **P Value** |
| **Age (Years)** |  |  |  |  |  |  |  |  |
| ≤25 | Ref. | - |  |  | Ref. | - | Ref. | - |
| 26 to 35 | 1.36 (0.76, 2.41) | 0.298 | - | - | 1.54 (0.91, 2.58) | 0.106 | 1.4 (0.78, 2.53) | 0.262 |
| 36 to 65 | 1.5 (0.79, 2.83) | 0.216 | - | - | 1.36 (0.76, 2.44) | 0.306 | 1.7 (0.85, 3.43) | 0.136 |
| **Gender** |  |  |  |  |  |  |  |  |
| Female | Ref. | - |  |  | Ref. | - |  |  |
| Male | 1.17 (0.75, 1.82) | 0.496 | - | - | 1 (0.65, 1.54) | 0.988 | - | - |
| **Residence** |  |  |  |  |  |  |  |  |
| Rural | Ref. | - | Ref. | - | Ref. | - | Ref. | - |
| Urban | 3.32 (1.73, 6.38) | 0.000 | 3.52 (1.73, 7.15) | 0.000 | 6.03 (1.82, 19.91) | 0.003 | 7.32 (1.97, 27.17) | 0.003 |
| **Educational Status** | |  |  |  |  |  |  |  |
| No Education | Ref. | - |  |  | Ref. | - | Ref. | - |
| Primary Completed | 0.82 (0.4, 1.7) | 0.595 | - | - | 2.2 (1.12, 4.32) | 0.022 | 2.19 (1.05, 4.58) | 0.037 |
| Secondary Completed | 0.67 (0.31, 1.45) | 0.304 | - | - | 3.47 (1.67, 7.18) | 0.001 | 2.76 (1.18, 6.41) | 0.019 |
| Higher | 0.97 (0.41, 2.33) | 0.949 | - | - | 6.02 (2.31, 15.73) | 0.000 | 4.67 (1.43, 15.27) | 0.011 |
| **Occupation** | |  |  |  |  |  |  |  |
| Service holder | Ref. | - | Ref. | - | Ref. | - | Ref. | - |
| Business | 0.92 (0.41, 2.08) | 0.847 | 1.16 (0.49, 2.71) | 0.740 | 1.82 (0.73, 4.5) | 0.198 | 2.93 (0.97, 8.83) | 0.056 |
| Farmer | 1.35 (0.64, 2.84) | 0.433 | 2.05 (0.91, 4.63) | 0.082 | 0.79 (0.36, 1.71) | 0.549 | 2.89 (1.05, 7.97) | 0.040 |
| Day Laborer | 0.5 (0.19, 1.3) | 0.156 | 0.73 (0.26, 1.99) | 0.534 | 0.39 (0.17, 0.92) | 0.032 | 1.18 (0.4, 3.55) | 0.762 |
| Rickshaw/Van Puller & Boat Driver | 0.71 (0.22, 2.26) | 0.558 | 1.12 (0.33, 3.74) | 0.860 | 1.58 (0.45, 5.5) | 0.473 | 4.41 (1.04, 18.83) | 0.045 |
| Housewife | 0.42 (0.18, 0.97) | 0.042 | 0.56 (0.24, 1.33) | 0.190 | 1.07 (0.49, 2.34) | 0.862 | 2.91 (1.08, 7.86) | 0.035 |
| Unemployed | 0.09 (0.01, 0.74) | 0.025 | 0.15 (0.02, 1.2) | 0.073 | 0.66 (0.24, 1.82) | 0.421 | 2.89 (0.86, 9.69) | 0.085 |
| Other | 0.77 (0.34, 1.74) | 0.528 | 1.1 (0.46, 2.61) | 0.829 | 1.3 (0.56, 3.02) | 0.547 | 3.54 (1.23, 10.18) | 0.019 |
| **Income Class (BDT)** | |  |  |  |  |  |  |  |
| <10,000 | Ref. | - |  |  | Ref. | - | Ref. | - |
| 10,000-20,000 | 0.95 (0.58, 1.55) | 0.830 | - | - | 1.09 (0.69, 1.71) | 0.711 | 0.9 (0.53, 1.54) | 0.711 |
| 20,001-50,000 | 1.31 (0.69, 2.5) | 0.405 | - | - | 6.85 (2.38, 19.74) | 0.000 | 6.16 (1.79, 21.21) | 0.004 |
| **Family Member** |  |  |  |  |  |  |  |  |
| ≤4 | Ref. | - |  |  | Ref. | - | Ref. | - |
| ≥5 | 1.14 (0.73, 1.78) | 0.557 | - | - | 0.41 (0.26, 0.65) | 0.000 | 0.39 (0.24, 0.64) | 0.000 |
