# Supplementary material for: Knowledge and acceptance of malaria vaccine among parents of under‐five children of malaria endemic areas in Bangladesh: A cross‐sectional study
Source: Health Expect. 2023 Sep 3;26(6):2630–43. doi: 10.1111/hex.13862 (PMC10632622; doi:10.1111/hex.13862)
Supplement: Supplementary file 2 — Supporting information. [file HEX-26--s001.docx]

### Supplementary table 2: Association of behavioral and household variables with Knowledge and Acceptance of Malaria Vaccine (N=405)

|  | **Knowledge Category** | | | | **Acceptance Category** | | | |
| --- | --- | --- | --- | --- | --- | --- | --- | --- |
| **Factor** | **Crude OR (95%CI)** | **P Value** | **Adj. OR (95%CI)** | **P Value** | **Crude OR (95%CI)** | **P Value** | **Adj. OR (95%CI)** | **P Value** |
| **Smoking Status** |  |  |  |  |  |  |  |  |
| No | Ref. | - | Ref. | - | Ref. | - | Ref. | - |
| Yes | 1.8 (1.12, 2.9) | 0.015 | 1.42 (0.83, 2.43) | 0.195 | 0.7 (0.44, 1.12) | 0.139 | 0.67 (0.35, 1.27) | 0.217 |
| **Comorbidity** |  |  |  |  |  |  |  |  |
| No | Ref. | - | Ref. | - | Ref. | - |  |  |
| Yes | 2.22 (1.26, 3.91) | 0.006 | 1.81 (0.94, 3.49) | 0.076 | 0.77 (0.43, 1.37) | 0.373 | - | - |
| **Bed Net** |  |  |  |  |  |  |  |  |
| No | Ref. | - |  |  | Ref. | - | Ref. | - |
| Yes | 0.7 (0.4, 1.23) | 0.217 | - | - | 1.48 (0.86, 2.56) | 0.156 | 1.31 (0.53, 3.22) | 0.555 |
| **Insecticide treated Bed Net** | |  |  |  |  |  |  |  |
| No | Ref. | - | Ref. | - | Ref. | - | Ref. | - |
| Yes | 1.37 (0.87, 2.15) | 0.179 | 0.82 (0.43, 1.56) | 0.540 | 0.31 (0.19, 0.49) | 0.000 | 0.42 (0.2, 0.9) | 0.026 |
| **Used Insecticide to Control Mosquito** | | | |  |  |  |  |  |
| No | Ref. | - | Ref. | - | Ref. | - | Ref. | - |
| Yes | 2.41 (1.51, 3.82) | 0.000 | 2.61 (1.41, 4.84) | 0.002 | 0.35 (0.22, 0.55) | 0.000 | 0.34 (0.16, 0.69) | 0.003 |
| **Housing Structure** |  |  |  |  |  |  |  |  |
| Ground Level House | Ref. | - |  |  | Ref. | - | Ref. | - |
| Stilted House | 1.15 (0.6, 2.19) | 0.672 | - | - | 2.19 (1.03, 4.65) | 0.041 | 2.27 (0.65, 7.97) | 0.200 |
| **House Wall** |  |  |  |  |  |  |  |  |
| Brick | Ref. | - |  |  | Ref. | - | Ref. | - |
| Cement | 0.64 (0.25, 1.63) | 0.347 | - | - | 1.49 (0.57, 3.9) | 0.420 | 1.98 (0.52, 7.51) | 0.316 |
| Tin | 1 (0.42, 2.39) | 0.994 | - | - | 2.74 (0.98, 7.63) | 0.054 | 13.02 (2.85, 59.48) | 0.001 |
| Wood | 3.18 (1.01, 9.97) | 0.048 | - | - | 1.33 (0.35, 5.04) | 0.672 | 0.5 (0.06, 3.96) | 0.510 |
| Bamboo | 0.77 (0.34, 1.77) | 0.537 | - | - | 0.44 (0.19, 1.01) | 0.054 | 0.93 (0.22, 4.03) | 0.924 |
| Mud | 0.98 (0.38, 2.5) | 0.958 | - | - | 0.46 (0.18, 1.17) | 0.104 | 1.56 (0.34, 7.13) | 0.566 |
| **House Floor** |  |  |  |  |  |  |  |  |
| Brick | Ref. | - | Ref. | - | Ref. | - | Ref. | - |
| Cement | 0.34 (0.23, 0.51) | 0.000 | 0 (0, Inf) | 0.986 | 8.92 (5.03, 15.83) | 0.000 | 4029793.84 (0, Inf) | 0.982 |
| Tin | 0.55 (0.18, 1.73) | 0.310 | 0.39 (0.12, 1.3) | 0.125 | 0.09 (0.03, 0.23) | 0.000 | 0.04 (0.01, 0.17) | 0.000 |
| Wood | 3.88 (1.5, 10.03) | 0.005 | 2.4 (0.82, 7.04) | 0.110 | 0.67 (0.17, 2.59) | 0.564 | 0.39 (0.07, 2.19) | 0.283 |
| Bamboo | 1.15 (0.62, 2.15) | 0.654 | 0.83 (0.4, 1.7) | 0.607 | 0.21 (0.1, 0.44) | 0.000 | 0.14 (0.03, 0.57) | 0.006 |
| Mud | 0.89 (0.51, 1.55) | 0.678 | 0.65 (0.34, 1.25) | 0.195 | 0.15 (0.08, 0.29) | 0.000 | 0.14 (0.04, 0.47) | 0.001 |
| **House Window** |  |  |  |  |  |  |  |  |
| Closable Window | Ref. | - | Ref. | - | Ref. | - | Ref. | - |
| No Window | 2.84 (1.52, 5.31) | 0.001 | 1.3 (0.59, 2.88) | 0.517 | 1.05 (0.57, 1.92) | 0.873 | 3.59 (1.47, 8.8) | 0.005 |
| Open Walls | 2.67 (1.6, 4.46) | 0.000 | 1.56 (0.79, 3.09) | 0.197 | 5.39 (2.67, 10.88) | 0.000 | 12.45 (4.27, 36.29) | 0.000 |
| **Awareness of Malaria** | |  |  |  |  |  |  |  |
| No | Ref. | - |  |  | Ref. | - | Ref. | - |
| Yes | 1.06 (0.55, 2.03) | 0.865 | - | - | 4.08 (2.28, 7.3) | 0.000 | 7.68 (3.33, 17.72) | 0.000 |
| **Ever been tested for Malaria** | | |  |  |  |  |  |  |
| No | Ref. | - | Ref. | - | Ref. | - | Ref. | - |
| Yes | 3.03 (1.91, 4.8) | 0.000 | 1.5 (0.84, 2.66) | 0.171 | 1.69 (1.08, 2.66) | 0.022 | 4.6 (2.17, 9.75) | 0.000 |
| **Ever been Diagnosed for Malaria** | | |  |  |  |  |  |  |
| No | Ref. | - | Ref. | - | Ref. | - | Ref. | - |
| Yes | 4.44 (2.73, 7.23) | 0.000 | 2.37 (1.28, 4.37) | 0.006 | 2.08 (1.2, 3.63) | 0.009 | 1.65 (0.7, 3.89) | 0.251 |
